# Adherence to low tidal volume in the transition to spontaneous ventilation in patients with acute respiratory failure in intensive care units in Latin America (SPIRAL): a study protocol

**DOI:** 10.62675/2965-2774.20240044-en

**Published:** 2024-07-23

**Authors:** Fabia Diniz-Silva, Bruno Valle Pinheiro, Luis Felipe Reyes, Alexandre Biasi Cavalcanti, Belinda Figueredo, Fernando Rios, Flávia Ribeiro Machado, Gabriel Preda, Guillermo Bugedo, Israel Silva Maia, Leda Tomiko Yamada da Silveira, Luis Herrera, Manuel Jibaja, Miguel Ibarra-Estrada, Mino Cestari, Nicolás Nin, Rollin Roldan, Tiago Mendonça dos Santos, Viviane Cordeiro Veiga, Alejandro Bruhn, Juliana Carvalho Ferreira, on behalf of SPIRAL Investigators, the Brazilian Research in Intensive Care Network (BRICNet), Latin America Intensive Care Network (LIVEN)

**Affiliations:** 1 Universidade de São Paulo Faculdade de Medicina Hospital das Clínicas São Paulo SP Brazil Division of Pulmonology, Instituto do Coração, Hospital das Clínicas, Faculdade de Medicina, Universidade de São Paulo - São Paulo (SP), Brazil.; 2 Universidade Federal de Juiz de Fora Hospital Universitário Juiz de Fora MG Brazil Hospital Universitário, Universidade Federal de Juiz de Fora - Juiz de Fora (MG), Brazil.; 3 Universidad de La Sabana Facultad de Medicina Unisabana Center for Translational Science Chia Colombia Unisabana Center for Translational Science, Facultad de Medicina, Universidad de La Sabana - Chia, Colombia.; 4 HCor-Hospital do Coração Research Institute São Paulo SP Brazil Research Institute, HCor-Hospital do Coração - São Paulo (SP), Brazil.; 5 Universidad Nacional de Asuncion Facultad de Ciencias Medicas Hospital de Clinicas Asuncion Paraguay Hospital de Clinicas, Facultad de Ciencias Medicas, Universidad Nacional de Asuncion - Asuncion, Paraguay.; 6 Hospital San Juan de Dios Ramos Mejia Argentina Hospital San Juan de Dios - Ramos Mejia, Buenos Aires, Argentina.; 7 Universidade Federal de São Paulo Department of Anesthesiology, Pain and Intensive Medicine São Paulo SP Brazil Department of Anesthesiology, Pain and Intensive Medicine, Universidade Federal de São Paulo - São Paulo (SP), Brazil.; 8 Sanatorio San Roque Asuncion Paraguay Sanatorio San Roque - Asuncion, Paraguay.; 9 Universidad Catolica de Chile Facultad de Medicina Department of Intensive Medicine Santiago Chile Department of Intensive Medicine, Facultad de Medicina, Pontificia Universidad Catolica de Chile - Santiago, Chile.; 10 Hospital Nereu Ramos Florianópolis SC Brazil Hospital Nereu Ramos, Florianópolis (SC), Brazil; 11 Universidade de São Paulo Hospital Universitário São Paulo SP Brazil Hospital Universitário, Universidade de São Paulo - São Paulo (SP), Brazil.; 12 Hospital IESS de Ibarra Ibarra Ecuador Hospital IESS de Ibarra - Ibarra, Ecuador.; 13 Universidad San Francisco Escuela Medicina Quito Ecuador Escuela Medicina, Universidad San Francisco - Quito, Ecuador.; 14 Hospital Civil Fray Antonio Alcalde Guadalajara Jalisco Mexico Hospital Civil Fray Antonio Alcalde - Guadalajara, Jalisco, Mexico.; 15 Hospital Alemão Oswaldo Cruz São Paulo SP Brazil Hospital Alemão Oswaldo Cruz - São Paulo (SP), Brazil.; 16 Hospital Espanhol Montevideo Uruguay Hospital Espanhol - Montevideo, Uruguay.; 17 Hospital Rebagliati Lima Peru Hospital Rebagliati - Lima, Peru.; 18 A Beneficência Portuguesa de São Paulo São Paulo SP Brazil BP - A Beneficência Portuguesa de São Paulo - São Paulo (SP), Brazil.

**Keywords:** Respiratory insufficiency, Respiration, artificial, Tidal volume, Oxygen, Incidence, Prevalence, Hospital mortality, Intensive care units

## Abstract

**Objective:**

Patients with acute respiratory failure often require mechanical ventilation to reduce the work of breathing and improve gas exchange; however, this may exacerbate lung injury. Protective ventilation strategies, characterized by low tidal volumes (≤ 8mL/kg of predicted body weight) and limited plateau pressure below 30cmH_2_O, have shown improved outcomes in patients with acute respiratory distress syndrome. However, in the transition to spontaneous ventilation, it can be challenging to maintain tidal volume within protective levels, and it is unclear whether low tidal volumes during spontaneous ventilation impact patient outcomes. We developed a study protocol to estimate the prevalence of low tidal volume ventilation in the first 24 hours of spontaneous ventilation in patients with hypoxemic acute respiratory failure and its association with ventilator-free days and survival.

**Methods:**

We designed a multicenter, multinational, cohort study with a 28-day follow-up that will include patients with acute respiratory failure, defined as a partial oxygen pressure/fraction of inspired oxygen ratio < 300mmHg, in transition to spontaneous ventilation in intensive care units in Latin America.

**Results:**

We plan to include 422 patients in ten countries. The primary outcomes are the prevalence of low tidal volume in the first 24 hours of spontaneous ventilation and ventilator-free days on day 28. The secondary outcomes are intensive care unit and hospital mortality, incidence of asynchrony and return to controlled ventilation and sedation.

**Conclusion:**

In this study, we will assess the prevalence of low tidal volume during spontaneous ventilation and its association with clinical outcomes, which can inform clinical practice and future clinical trials.

## INTRODUCTION

Patients with acute respiratory failure (ARF) generally require the use of mechanical ventilation (MV) to reduce the work of breathing and maintain adequate gas exchange. However, experimental and clinical studies have demonstrated that MV can contribute to worsening lung injury.^(1,2)^ Clinical trials have shown that the use of protective ventilation, which consists of using tidal volumes (V_T_) equal to or less than 8mL/kg of predicted body weight (PBW) and limiting airway plateau pressure below 30cmH_2_O, is associated with better clinical results and reduced mortality in patients with acute respiratory distress syndrome (ARDS).^(3–7)^

In the initial phase of MV, patients are frequently ventilated in assisted-controlled modes, in which tight control of V_T_ and/or airway plateau pressure is more easily implemented. However, controlled ventilation usually requires sedation and sometimes the use of neuromuscular blockers.^(8,9)^ Previous studies have shown that the use of controlled ventilation is associated with reduced diaphragmatic muscle mass with consequent muscle weakness.^(10–13)^ Conversely, overload of the respiratory muscles during the acute phase of respiratory failure can cause fatigue and is associated with respiratory muscle injury.^(14)^

One way to avoid these two extremes is to use assisted ventilation modes in which the patient's spontaneous inspiratory efforts are supported by the ventilator, avoiding disuse of the respiratory muscles,^(10–12)^ without generating fatigue, since the patient is supported by the ventilator during inspiration.

However, the management of the patient's inspiratory effort presents an important clinical challenge, as patients with acute lung injury may have high levels of respiratory drive, which can result in high V_T_ and/or patient ventilator asynchrony. The development of strategies to regulate respiratory drive may allow a safe compromise between the benefits and risks of spontaneous breathing during MV.^(15)^

Despite the strong scientific evidence supporting protective ventilation, several studies have identified low adherence to it and found significant barriers to the implementation of a protective ventilation strategy in patients with ARF and ARDS.^(16–18)^ A large observational study that included patients in 50 countries revealed an association between low V_T_ and better survival^(19)^ but low adherence to low V_T_. This study was performed before the coronavirus disease 2019 (COVID-19) pandemic, which mobilized the world to manage a large number of ventilated patients, which might have increased the knowledge of how to manage patients with ARDS under MV. Clinical guidelines recommend protective ventilation for COVID-19 patients, and studies conducted during the pandemic have shown greater adherence to the use of low V_T_ during controlled MV and an association with better survival.^(20,21)^ However, observational studies conducted in COVID-19 and non-COVID-19 patients have evaluated adherence to protective ventilatory strategies in the early days of MV, during which patients are typically in assist-control ventilatory modes. To what extent adherence to protective ventilatory parameters occurs during spontaneous ventilation remains unknown. Moreover, it is unknown how much ventilatory practices changed in the aftermath of the COVID-19 pandemic.

There are no studies evaluating the use of low V_T_ for spontaneous ventilation in patients with ARF in Latin America. This study aims to assess the prevalence of low V_T_ ventilation implementation in the first 24 hours of spontaneous ventilation in intensive care units (ICUs) in Latin America and its association with important clinical outcomes.

## METHODS

### Study design and location

This multicenter, multinational, cohort study protocol will include patients with ARF transitioning to spontaneous ventilation in ICUs in Latin American countries.

We will invite investigators from ICUs in ten Latin American countries. Invitations will be sent to all ICUs in the Brazilian Research in Intensive Care Network (BRICNet) and Latin American Intensive Care Network (LIVEN) databases by email, and we have identified one investigator in each of the countries to serve as a national coordinator responsible for using a snowball strategy to invite ICUs and support the local investigators with regulatory and logistic issues.

### Objectives and outcomes

The primary objectives are to estimate the prevalence of low V_T_ ventilation (V_T_ < 8mL/kg of PBW) in the first 24 hours of spontaneous ventilation in patients with hypoxemic ARF and its association with ventilator-free days on day 28. Tidal volume will be assessed five times over the first 24 hours after the transition to spontaneous ventilation to account for potential variation. To determine whether V_T_ was below or above 8 mL/kg of PBW for a given patient, we will calculate the weighted average V_T_ based on the duration of each evaluation period. Accordingly, the first assessment, collected one hour after the transition, carries a weight of 1/24, the second assessment, collected 5 hours after (and 6 hours after the transition), carries a weight of 5/24, and the third, fourth and fifth assessments, collected after 12 hours, 18 hours and 24 hours of the transition, respectively, will each carry a weight of 6/24.

The secondary objectives are to estimate the association between the rate of adherence to low V_T_ ventilation in spontaneous ventilation and survival, estimate the incidence of asynchrony in the first 24 hours after the transition to spontaneous ventilation and associated mortality, estimate the proportion of patients who returned to sedation and controlled MV in the first 24 hours or at any time after the transition to spontaneous ventilation, assess the adherence to low positive end-expiratory pressure (PEEP) by fraction of inspired oxygen (FiO_2_) table in the first 24 hours after the transition to spontaneous ventilation, and identify barriers associated with nonadherence to low V_T_ ventilation during spontaneous ventilation.

### Eligibility criteria

**Figure 1:**
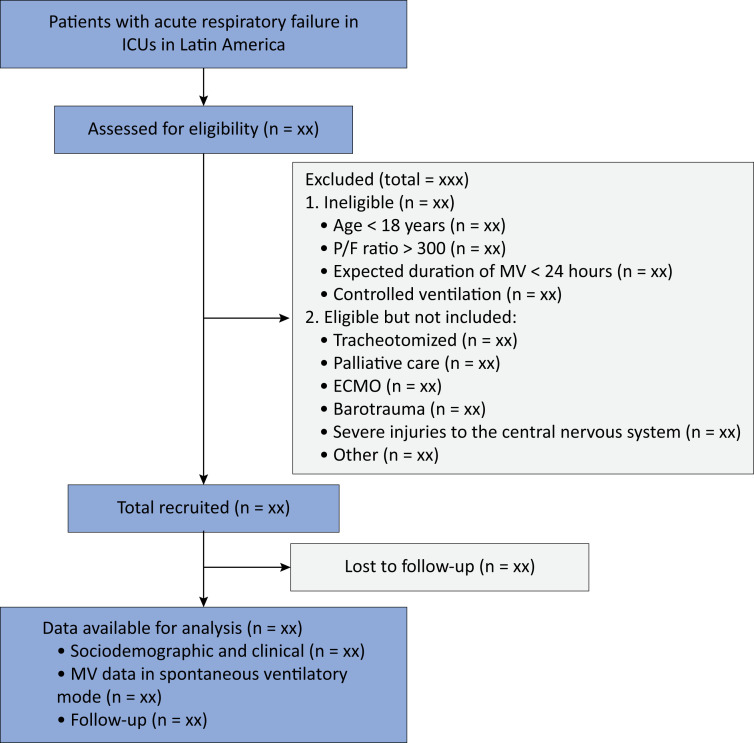
Study flow diagram.

We will screen all patients admitted to the participating ICUs under MV and include patients who meet the eligibility criteria (Figure 1).

ICU - intensive care unit; P/F - partial pressure of oxygen and fraction of inspired oxygen ratio; MV - mechanical ventilation; ECMO - extracorporeal membrane oxygenation.

The inclusion and exclusion criteria are described in table 1.

**Table 1:** Participant inclusion and exclusion criteria

Inclusion criteria
• Age > 18 years.
• Patients with hypoxemic ARF, defined as a PaO_2_/FiO_2_ ratio ≤ 300mmHg, under controlled invasive MV, with an expected duration of MV of at least 24 hours.
• Patients transitioned to spontaneous ventilation (PSV, PAV Plus, NAVA, CPAP and APRV).
**Exclusion criteria**
• Tracheostomized patients.
• Decision not to maintain or add life support measures on the day of assessment (palliative care).
• Patients in ECMO.
• Patients with an air fistula or barotrauma that prevents adequate tidal volume monitoring.
• Severe injuries to the central nervous system that result in abolished or very high respiratory drive, for whom it is not possible to maintain a protective tidal volume.

ARF - acute respiratory failure; PaO_2_/FiO_2_ - partial oxygen pressure/fraction of inspired oxygen; MV - mechanical ventilation; PSV - pressure support ventilation; PAV - proportional assist ventilation; NAVA - neurally adjusted ventilatory assist; CPAP - continuous positive airway pressure; APRV - airway pressure release ventilation; ECMO - extracorporeal membrane oxygenation.

### Sample size calculation

There are no previous studies that have estimated the rate of adherence to low V_T_ in patients with spontaneous ventilation; therefore, we used a conservative approach. To estimate the prevalence with a confidence limit of 5% precision, an anticipated adherence of 50%, and up to 10% loss to follow-up, we estimated that we need to include 422 patients. Additionally, we calculated the power to detect a 3 day difference in ventilator-free days with a standard deviation of 9 days,^(22)^ and we anticipate a statistical power of at least 80% even with a loss to follow-up rate of 10%, provided that the prevalence is between 25 and 75%.

### Study measures and data collection

The following baseline characteristics related to admission to the ICU and initiation of MV will be obtained from the patient's medical records: age, sex, height (assessed using a tape measure with the patient in the supine position), weight reported by the ICU team, main reason for admission to the ICU, cause of ARF, arterial oxygen pressure/fraction of inspired oxygen ratio (PaO_2_/FiO_2_) on the day of the ARF diagnosis, date of intubation, risk factors for ARDS, comorbidities (cardiac, respiratory, neurological, renal, hepatic, immunosuppression, cancer, arterial hypertension, diabetes and smoking) and Simplified Acute Physiology Score 3 (SAPS 3). We will calculate the PBW according to the following formulas:


$$\begin{array}{c} 50+0.91\times \left(\text{height in centimeters}-\;152.4\right)\text{for males}\\ 45.5+0.91\times \left(\text{height in centimeters}-\;152.4\right)\text{for females} \end{array}$$

We will also retrospectively collect the ventilatory parameters from the day before inclusion in the study from the patient's medical records, in addition to the use of vasopressors, sedatives, neuromuscular blockers and analgesics, ARDS diagnosis, Richmond Agitation-Sedation Scale (RASS) score, presence of *delirium* assessed by the Confusion Assessment Method in an Intensive Care Unit (CAM-ICU) scale or clinical assessment, Sepsis-related Organ Failure (SOFA) score, blood gas analysis and other laboratory data for diagnosing organ failure. We will also calculate the respiratory system compliance and resistance from the day before study enrollment (for patients under deep sedation and/or neuromuscular blockage). The main study measures and data collection methods are shown in table 2.

**Table 2:** Characteristics of the study population

Characteristic		Value
Age (years)		xx ± x
Male, n (%)		n (xx)
BMI (kg/m^2^)		xx ± x
SAPS 3 score		xx ± x
SOFA		xx (xx - xx)
LIP score		xx (xx - xx)
Charlson index		xx (xx - xx)
Cause ARF		
	Pneumonia (bacterial, viral, opportunistic)	n (xx)
	Extrapulmonary sepsis	n (xx)
	Coma/lowered level of consciousness	n (xx)
	Cardiogenic pulmonary edema/heart failure	n (xx)
	Aspiration	n (xx)
	Hypovolemic shock	n (xx)
	Others	n (xx)
Days of intubation prior to inclusion		xx (xx - xx)
Cause of hospitalization		
	Acute respiratory failure	n (xx)
	Sepsis	n (xx)
	Neurologic diseases	n (xx)
	Postoperative monitoring	n (xx)
PaO_2_/FiO_2_ ratio at ARF diagnosis		xx ± x
Duration of invasive MV (days)		xx (xx - xx)
Mechanical ventilation-free days (days)		xx (xx - xx)
Duration of ICU stay (days)		xx (xx - xx)
ICU survival, n (%) [95%CI]		n (xx) (xx- xx)
Duration of hospital stay (days)		xx (xx - xx)
Hospital survival, n (%) [95%CI]		n (xx) (xx- xx)

BMI - body mass index; SAPS3 - Simplified Acute Physiology Score 3; SOFA - Sequential Organ Failure Assessment; LIP - lung injury prediction; ARF - acute respiratory failure; PaO_2_/FiO_2_ - partial oxygen pressure/fraction of inspired oxygen; MV - mechanical ventilation; IQR - interquartile rage; ICU - intensive care unit. The data are expressed as the mean ± standard deviation, n (%), median (interquartile range) or n (%) (95%CI).

### Data collection on the day of transition to spontaneous ventilation

On the day of enrollment in the study, we will collect ventilatory parameters one hour before the transition to spontaneous ventilation and periodically during the first 24 hours of spontaneous ventilation. We will include patients who are transitioned and remain for at least one hour in one of the following ventilatory modes: pressure support ventilation (PSV), continuous positive airway pressure (CPAP), airway pressure release ventilation (APRV), neurally adjusted ventilatory assist (NAVA), proportional assist ventilation (PAV +) or adaptive ventilatory assist (AVA).

### One hour before transition

The following data on controlled ventilation will be recorded retrospectively: ventilatory mode; programmed respiratory rate; patient's total respiratory rate; minute ventilation; peak airway pressure; V_T_; and PEEP. We will also collect vital data (heart rate, mean arterial pressure, peripheral oxygen saturation), arterial blood gas analysis data (obtained as close as possible to the collection of ventilatory data will be recorded), the presence of major asynchronies,^(23)^ the use of vasopressors, the use of sedatives and analgesics, the RASS score, the presence of *delirium* assessed by the CAM-ICU scale or clinical assessment.

### One hour and every 6 hours until 24 hours after transition to spontaneous ventilation

Once the patient is included in the study, we will prospectively collect data at the end of the first hour and every six hours (with a tolerance of ± 1 hour) after the transition, including the ventilatory mode, patient's respiratory rate, minute ventilation, peak airway pressure, V_T_, PEEP, sensitivity setting, airway occlusion pressure at 100ms (P 0.1) and presence of asynchronies. We will also collect vital data, arterial blood gas analysis (if available), data on the use of vasopressors, data on the use of sedatives and analgesics, and data on sedation levels measured by the RASS and the presence of *delirium* (Table 3).

**Table 3:** Data from the day of inclusion in the study

Variable	1 hour before	1 hour after	6 hours after	12 hours after	18 hours after	24 hours after
Tidal volume (mL/kg of PBW)	xx ± xx	xx ± xx	xx ± xx	xx ± xx	xx ± xx	xx ± xx
PEEP (cmH_2_O)	xx ± xx	xx ± xx	xx ± xx	xx ± xx	xx ± xx	xx ± xx
Respiratory rate (breaths/min)	xx ± xx	xx ± xx	xx ± xx	xx ± xx	xx ± xx	xx ± xx
Ppeak, (cmH_2_O)	xx ± xx	xx ± xx	xx ± xx	xx ± xx	xx ± xx	xx ± xx
Minute ventilation (liters/min)	xx ± xx	xx ± xx	xx ± xx	xx ± xx	xx ± xx	xx ± xx
FiO_2_	xx ± xx	xx ± xx	xx ± xx	xx ± xx	xx ± xx	xx ± xx
pH	xx ± xx	xx ± xx	xx ± xx	xx ± xx	xx ± xx	xx ± xx
PaO_2_ (mmHg)	xx ± xx	xx ± xx	xx ± xx	xx ± xx	xx ± xx	xx ± xx
SaO_2_	xx ± xx	xx ± xx	xx ± xx	xx ± xx	xx ± xx	xx ± xx
Heart rate (bpm)	xx ± xx	xx ± xx	xx ± xx	xx ± xx	xx ± xx	xx ± xx
Mean arterial pressure (mmHg)	xx ± xx	xx ± xx	xx ± xx	xx ± xx	xx ± xx	xx ± xx
RASS	x (x - x)	x (x - x)	x (x - x)	x (x - x)	x (x - x)	x (x - x)
*Delirium*	x (x)	x (x)	x (x)	x (x)	x (x)	x (x)
Asynchrony	x (x)	x (x)	x (x)	x (x)	x (x)	x (x)
Vasopressors	x (x)	x (x)	x (x)	x (x)	x (x)	x (x)

PBW - predicted body weight; PEEP - positive end-expiratory pressure; Ppeak - peak airway pressure; FiO_2_ - fraction of inspired oxygen; pH - potential of hydrogen; PaO_2_ - partial pressure of oxygen; SaO_2_ - oxygen saturation of arterial blood; RASS - Richmond Agitation and Sedation Scale. The data are expressed as the mean ± standard deviation, median (interquartile range) or n (%).

### Follow-up

We will follow patients until hospital discharge or death within the hospital and will compute the rate of adherence to low V_T_, that is, V_T_ ≤ 8mL/kg of PBW in the first 24 hours of ventilation in spontaneous ventilation, the proportion of patients with significant asynchrony in the transition to spontaneous MV, the proportion of patients returning for sedation and controlled MV in the first 24 hours, the proportion of patients returning to sedation and controlled MV at any time during MV, the rate of adherence to a low PEEP/FiO_2_ table in the first 24 hours, 28 day hospital survival and ventilator-free days. Ventilator-free days will be calculated as the number of days alive and out of MV to day 28. Patients who die before day 28 will be considered to have zero ventilator-free days; the day of inclusion will be considered day 0; for patients who are extubated and reintubated in the first 28 days, the number of ventilator-free days will be computed as the number of days alive and out of MV from the day of the last successful extubation (> 48 hours without MV) to day 28; for tracheostomized patients, the number of ventilator-free days will be computed as the number of days alive and out of MV from the day that the patient remains disconnected from the MV for more than six consecutive hours to day 28.

**Figure 2:**
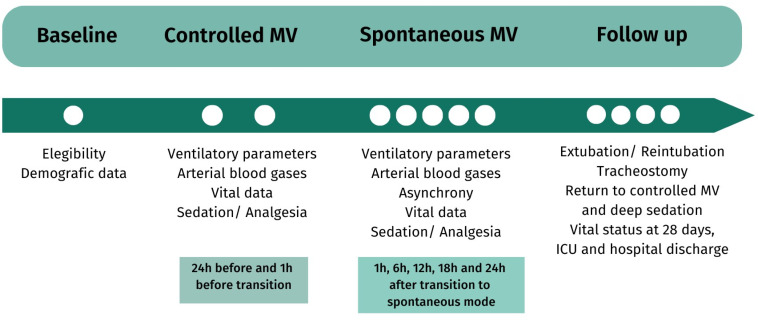
Data collection schedule.

The data collection schedule is shown in figure 2 and table 1S (Supplementary Material).

MV - mechanical ventilation; ICU - intensive care unit.

### Statistical plan analysis

We will perform descriptive statistics, using the mean and standard deviation for continuous variables that present a normal distribution, the median and interquartile ranges for continuous variables that do not present a normal distribution and proportions for categorical variables.

Differences between patients receiving V_T_ less than or equal to 8mL/kg of PBW and patients receiving greater than 8mL/kg of PBW in the first 24 hours after transition to spontaneous ventilation will be evaluated using t tests, Mann–Whitney tests or chi–square tests, according to patient characteristics and data distribution.

The prevalence of adherence to low V_T_ ventilation in spontaneous ventilation during the initial 24 hours (V_T_ less than 8mL/kg of PBW), proportion of asynchrony in the first 24 hours after the transition to spontaneous ventilation, proportion of patients who return to sedation and controlled MV in the first 24 hours or at any time after the transition to spontaneous ventilation and adherence to a low PEEP/FiO_2_ table in the first 24 hours after the transition to spontaneous ventilation will be presented, along with the respective 95% confidence intervals.

Possible barriers associated with nonadherence (V_T_ during the initial 24 hours of spontaneous ventilation less than 8mL/kg of PBW) will be analyzed using a logistic regression model.

To characterize hospital survival at 28 days based on V_T_ during the initial 24 hours of spontaneous ventilation, we will employ the Cox proportional hazards model and generate Kaplan–Meier curves. We will analyze mortality within 28 days using the V_T_ during the initial 24 hours of spontaneous ventilation and according to the incidence of asynchrony during the first 24 hours after the transition to spontaneous ventilation using mixed logistic regressions with the center treated as a random effect.

The comparison of V_T_ during the initial 24 hours of spontaneous ventilation (less than 8mL/kg of PBW versus greater than 8mL/kg) in relation to ventilator-free days within 28 days will be performed using mixed generalized regression models considering the distribution that best fits the data, incorporating center as a random effect. We constructed a conceptual causal diagram in the format of a directed acyclic graph (DAG),^(24)^ including the association between V_T_ during the first 24 hours of spontaneous ventilation and ventilator-free days at 28 days and the most relevant variables (Figure 1S - Supplementary Material): body mass index (BMI) and SAPS 3 at baseline, SOFA on the day before transition, PaO_2_/FiO_2_ at the moment of the ARF diagnosis, and PEEP, RASS, and pH one hour before transition.

All analyses will be performed using the R statistical program (R Core Team; Vienna, Austria; https://www.R-project.org), and we will consider p < 0.05 to indicate statistical significance.

### Data collection and quality assessment

All data will be collected at each center by an investigator trained in an electronic case report form (CRF) developed in the Research Electronic Data Capture (RedCap) electronic database. The electronic database has its fields formatted to accept specific ranges of values for each variable, reducing the chances of entering incorrect values. The most important variables will be analyzed for missing, discrepant or inconsistent data. A backup copy of the database will be saved periodically and will be archived at the end of the study.

### Ethical aspects

The study was approved by the Research Ethics Committee of the *Hospital das Clínicas* of the *Universidade de São Paulo* (CAAE 28482720.0.1001.0068) and by the Ethics Committee of each participating institution, when needed. Informed consent was waived due to the observational nature of the study. The protocol is registered on the international platform ClinicalTrials.gov (NCT06042036). Each patient will be identified by a study number to protect confidentiality.

We will submit the study results for publication in accordance with the recommended guidelines for reporting observational studies, The Strengthening the Reporting of Observational Studies in Epidemiology (Strobe).^(25)^ The results of the primary study will be published in a peer-reviewed journal.

### Current status

The recruitment of participants started in June 2023, and we expect to complete this study by June 2024.

## DISCUSSION

The SPIRAL study was developed to describe the ventilation of patients with ARF in spontaneous ventilation, mainly with respect to the V_T_ applied in this phase. Randomized clinical trials have shown that low V_T_ and limited plateau pressure during controlled ventilation reduce mortality and ventilator-induced lung injury,^(3,7)^ and clinical practice guidelines recommend using protective ventilation, defined as a V_T_ of 4 - 8mL/kg and a plateau pressure below 30cmH_2_O.^(2,26)^ However, in spontaneous ventilation, the V_T_ varies according to the adjusted level of inspiratory assistance and the patient's inspiratory effort, which makes it difficult to control the V_T_. Previous physiological studies^(27,28)^ have shown that it is possible to maintain V_T_ below 8mL/kg of PBW in the majority of patients diagnosed with ARDS who are transitioning to spontaneous ventilation. The study's focus on adherence to low V_T_ during spontaneous ventilation reflects challenges faced by clinicians in daily practice. We chose to use a cutoff of 8mL/kg PBW instead of 6mL/kg because although most clinical randomized trials have targeted 6mL/kg PBW, V_T_ up to 8mL/kg were allowed in most studies.^(4,7)^ Additionally, clinical guidelines define protective ventilation as a V_T_ of 4 - 8 mL/kg^(2,26)^ and prior observational trials^(19)^ have used this definition.

The study's primary and secondary objectives cover a broad spectrum of clinical outcomes, including ventilator-free days, survival, incidence of asynchrony, and identification of potential barriers to adherence to low V_T_, enabling a comprehensive view of the impact of ventilation strategies on patient-centered outcomes. Data will be collected at several timepoints during the first 24 hours after the transition to spontaneous ventilation. This approach takes into account possible variations over time and will enable a differentiated understanding of V_T_ dynamics during this critical period. Due to possible variations in V_T_ with spontaneous ventilation over time, we chose to use the weighted average of V_T_ to obtain a more representative assessment of the ventilation dynamics during the first 24 hours of spontaneous ventilation.

The multicenter and multinational characteristics of our study, with the participation of ten Latin American countries, add significant diversity to the population of patients included and allow a more comprehensive understanding of regional variations in clinical practices. This observational study will reflect real-life clinical scenarios, and the findings will be derived from routine patient care settings, increasing the practical relevance of the results. This study has the potential to influence clinical decision-making, inform future clinical trials, and ultimately improve patient outcomes.

Our study has some limitations. We will collect detailed data regarding ventilatory parameters only for the first 24 hours of the transition to spontaneous ventilation, and we recognize that these first 24 hours are not necessarily representative of the remaining days in MV; in addition, patients can only be included once in the study because survival is one of the secondary outcomes; therefore, we will only collect data for the first transition. The tidal volume will not be obtained continuously but rather will be collected at several timepoints during the first 24 hours of spontaneous ventilation. To better represent these variations, we will calculate the weighted average of the V_T_ based on the duration of each evaluation period. This method takes into account changes in V_T_ over a 24-hour period, assigning different weights to each data point.

The participating ICUs in the study were invited using the databases of large research networks, and it is possible that the investigators in the ICUs had more knowledge and experience with MV than did the average Latin American ICU practitioners, which could generate selection bias and restrict the external validity of the study. However, invitations were sent to a large number of ICUs regardless of research experience, and we did not restrict participation to active network members. Although the multinational approach increases external validity, the specific regional characteristics of Latin American ICUs may limit the generalizability of the results to countries in other parts of the world.

In conclusion, this study may provide valuable contributions to understanding protective ventilation practices in patients with ARF during the transition to spontaneous ventilation in Latin American ICUs, especially after the COVID-19 pandemic. The results can potentially inform clinical practice and future clinical trials and impact the treatment of patients with acute respiratory failure.
