# Supplementary material for: Adherence to low tidal volume in the transition to spontaneous ventilation in patients with acute respiratory failure in intensive care units in Latin America (SPIRAL): a study protocol
Source: Crit Care Sci. 2024 Jul 23;36:e20240044en. doi: 10.62675/2965-2774.20240044-en (PMC11321717; doi:10.62675/2965-2774.20240044-en)
Supplement: Translation

# Translation: Aderência ao volume corrente baixo na transição para ventilação espontânea em pacientes com insuficiência respiratória aguda em unidades de terapia intensiva na América Latina (SPIRAL): um protocolo de estudo

**DOI:** 10.62675/2965-2774.20240044-pt

**Authors:** Fabia Diniz-Silva, Bruno Valle Pinheiro, Luis Felipe Reyes, Alexandre Biasi Cavalcanti, Belinda Figueredo, Fernando Rios, Flávia Ribeiro Machado, Gabriel Preda, Guillermo Bugedo, Israel Silva Maia, Leda Tomiko Yamada da Silveira, Luis Herrera, Manuel Jibaja, Miguel Ibarra-Estrada, Mino Cestari, Nicolás Nin, Rollin Roldan, Tiago Mendonça dos Santos, Viviane Cordeiro Veiga, Alejandro Bruhn, Juliana Carvalho Ferreira, em nome de SPIRAL Investigators, the Brazilian Research in Intensive Care Network (BRICNet), Latin America Intensive Care Network (LIVEN)

**Affiliations:** 1 São Paulo SP Brasil Divisão de Pneumologia, Instituto do Coração, Hospital das Clínicas, Faculdade de Medicina, Universidade de São Paulo - São Paulo (SP), Brasil.; 2 Juiz de Fora MG Brasil Hospital Universitário, Universidade Federal de Juiz de Fora - Juiz de Fora (MG), Brasil.; 3 Chia Colômbia Unisabana Center for Translational Science, Facultad de Medicina, Universidad de La Sabana - Chia, Colômbia.; 4 São Paulo SP Brasil Instituto de Pesquisa, HCor-Hospital do Coração - São Paulo (SP), Brasil.; 5 Assunção Paraguai Hospital de Clinicas, Facultad de Ciencias Medicas, Universidad Nacional de Asuncion - Assunção, Paraguai.; 6 Ramos Mejia Argentina Hospital San Juan de Dios - Ramos Mejia, Buenos Aires, Argentina.; 7 São Paulo SP Brasil Departamento de Anestesiologia, Dor e Terapia Intensiva, Universidade Federal de São Paulo - São Paulo (SP), Brasil.; 8 Assunção Paraguai Sanatorio San Roque - Assunção, Paraguai.; 9 Santiago Chile Departamento de Medicina Intensiva, Facultad de Medicina, Pontificia Universidad Catolica de Chile - Santiago, Chile.; 10 Florianópolis SC Brasil Hospital Nereu Ramos, Florianópolis (SC), Brasil; 11 São Paulo SP Brasil Hospital Universitário, Universidade de São Paulo - São Paulo (SP), Brasil.; 12 Ibarra Equador Hospital IESS de Ibarra - Ibarra, Equador.; 13 Quito Equador Escuela Medicina, Universidad San Francisco - Quito, Equador.; 14 Guadalajara Jalisco México Hospital Civil Fray Antonio Alcalde - Guadalajara, Jalisco, México.; 15 São Paulo SP Brasil Hospital Alemão Oswaldo Cruz - São Paulo (SP), Brasil.; 16 Montevidéu Uruguai Hospital Espanhol - Montevidéu, Uruguai.; 17 Lima Peru Hospital Rebagliati - Lima, Peru.; 18 São Paulo SP Brasil BP - A Beneficência Portuguesa de São Paulo - São Paulo (SP), Brasil.

**Keywords:** Insuficiência respiratória, Respiração artificial, Volume de ventilação pulmonar, Oxigênio, Incidência, Prevalência, Mortalidade hospitalar, Unidades de terapia intensiva

## Abstract

**Objetivo:**

Pacientes com insuficiência respiratória aguda frequentemente necessitam de ventilação mecânica para reduzir o trabalho respiratório e melhorar a troca gasosa; no entanto, a ventilação mecânica pode exacerbar a lesão pulmonar. As estratégias de ventilação protetora, caracterizadas por baixo volume corrente (≤ 8mL/kg de peso corporal previsto) e pressão de platô limitada abaixo de 30cmH_2_O, mostraram melhores desfechos em pacientes com síndrome do desconforto respiratório agudo. No entanto, na transição para a ventilação espontânea, manter o volume corrente dentro dos níveis de proteção pode ser desafiador, e não está claro se o volume corrente baixo durante a ventilação espontânea afeta os desfechos dos pacientes. Desenvolvemos um protocolo de estudo para estimar a prevalência de ventilação com volume corrente baixo nas primeiras 24 horas de ventilação espontânea em pacientes com insuficiência respiratória aguda hipoxêmica e sua associação com dias livres da ventilação mecânica e sobrevida.

**Métodos:**

Desenhamos um estudo de coorte multicêntrico, multinacional, com seguimento de 28 dias, que incluirá pacientes com insuficiência respiratória aguda, definida como pressão parcial de oxigênio/fração inspirada de oxigênio < 300mmHg, em transição para ventilação espontânea em unidades de terapia intensiva na América Latina.

**Resultados:**

Pretendemos incluir 422 pacientes em 10 países. Os desfechos primários são a prevalência de baixo volume corrente nas primeiras 24 horas de ventilação espontânea e dias livres da ventilação mecânica no dia 28. Os desfechos secundários são mortalidade hospitalar e na unidade de terapia intensiva, incidência de assincronia e retorno à ventilação controlada e sedação.

**Conclusão:**

Neste estudo, avaliaremos a prevalência de volume corrente baixo durante a ventilação espontânea e sua associação com os desfechos clínicos, o que pode informar a prática clínica e futuros ensaios clínicos.

## INTRODUÇÃO

Pacientes com insuficiência respiratória aguda (IRpA) geralmente requerem o uso de ventilação mecânica (VM) para reduzir o trabalho respiratório e manter a troca gasosa adequada. No entanto, estudos experimentais e clínicos demonstraram que a VM pode contribuir para o agravamento da lesão pulmonar.^(1,2)^ Ensaios clínicos demonstraram que o uso de ventilação protetora, em que se utilizam volume corrente (VC) igual ou inferior a 8mL/kg de peso corporal previsto (PCP) e pressão de platô das vias aéreas limitada a menos de 30cmH_2_O, associa-se a melhores resultados clínicos e menor mortalidade em pacientes com síndrome do desconforto respiratório agudo (SDRA).^(3–7)^

Na fase inicial da VM, os pacientes são frequentemente ventilados em modos assistidos-controlados, nos quais é mais fácil implementar um controle rígido do VC e/ou da pressão de platô das vias aéreas. No entanto, a ventilação controlada geralmente requer sedação e, às vezes, o uso de bloqueadores neuromusculares.^(8,9)^ Estudos anteriores mostraram que o uso de ventilação controlada está associado à redução da massa muscular diafragmática com consequente fraqueza muscular.^(10–13)^ Por outro lado, a sobrecarga dos músculos respiratórios durante a IRpA pode causar fadiga e está associada à lesão da musculatura respiratória.^(14)^

Uma forma de evitar esses dois extremos é utilizar modos de ventilação assistida em que os esforços inspiratórios espontâneos do paciente são assistidos pelo ventilador, evitando o desuso da musculatura respiratória,^(10–12)^ sem gerar fadiga, uma vez que o paciente recebe suporte do ventilador durante a inspiração.

No entanto, o manejo do esforço inspiratório representa um importante desafio clínico, pois os pacientes com lesão pulmonar aguda podem apresentar altos níveis de *drive* respiratório, o que pode resultar em VC elevado e/ou assincronia paciente-ventilador. O desenvolvimento de estratégias para regular o *drive* respiratório possibilita um ajuste seguro entre os benefícios e os riscos da respiração espontânea durante a VM.^(15)^

Apesar das fortes evidências científicas que apoiam a ventilação protetora, diversos estudos identificaram sua baixa adesão e encontraram obstáculos significativos para a implementação de uma estratégia de ventilação protetora em pacientes com IRpA e SDRA.^(16–18)^ Em um grande estudo observacional que incluiu pacientes de 50 países, constatou-se uma associação entre VC baixo e maior sobrevida,^(19)^ mas baixa adesão ao VC baixo. Esse estudo foi realizado antes da pandemia da doença pelo coronavírus 2019 (COVID-19), a qual mobilizou a comunidade mundial no manejo de um número considerável de pacientes ventilados, o que pode ter aumentado o conhecimento sobre o manejo de pacientes com SDRA sob VM. As diretrizes clínicas recomendam a ventilação protetora em pacientes com COVID-19, e estudos realizados durante a pandemia mostraram maior adesão ao uso de VC baixo durante a VM controlada e associação com maior sobrevida.^(20,21)^ No entanto, estudos observacionais realizados em pacientes com e sem COVID-19 avaliaram a adesão às estratégias ventilatórias protetoras nos primeiros dias de VM, durante os quais os pacientes normalmente estão em modos ventilatórios assistido-controlado. Ainda não se sabe até que ponto a aderência a parâmetros ventilatórios protetores ocorre durante a ventilação espontânea. Além disso, não se sabe o quanto as práticas ventilatórias mudaram após a pandemia da COVID-19.

Não há estudos que avaliem o uso de VC baixo na ventilação espontânea em pacientes com IRpA na América Latina. Este estudo tem como objetivo avaliar a prevalência da implementação da ventilação com VC baixo nas primeiras 24 horas de ventilação espontânea em unidades de terapia intensiva (UTIs) na América Latina e sua associação com desfechos clínicos importantes.

## MÉTODOS

### Desenho e local do estudo

Trata-se de protocolo de estudo multicêntrico, multinacional e de coorte, que incluirá pacientes com IRpA em transição para a ventilação espontânea em UTIs de países da América Latina.

Convidaremos pesquisadores de UTIs de dez países latino-americanos. Os convites serão enviados a todas as UTIs dos bancos de dados da Rede Brasileira de Pesquisa em Terapia Intensiva (BRICNet) e *Latin American Intensive Care Network* (LIVEN) por *e-mail*. Identificamos um pesquisador em cada um dos países para atuar como coordenador nacional, responsável por usar uma estratégia de bola de neve para convidar UTIs e prestar apoio aos pesquisadores locais em questões regulatórias e logísticas.

### Objetivos e desfechos

Os objetivos primários são estimar a prevalência de ventilação com VC baixo (VC < 8mL/kg de PCP) nas primeiras 24 horas de ventilação espontânea em pacientes com IRpA hipoxêmica e sua associação com dias livres da VM no dia 28. O VC será avaliado cinco vezes nas primeiras 24 horas após a transição para a ventilação espontânea para considerar a possível variação. De modo a determinar se o VC de determinado paciente estava abaixo ou acima de 8mL/kg de PCP, calcularemos a média ponderada do VC em função da duração de cada período de avaliação. Dessa forma, a primeira avaliação, coletada 1 hora após a transição, tem peso de 1/24, a segunda avaliação, coletada 5 horas após (e 6 horas após a transição), tem peso de 5/24, e a terceira, quarta e quinta avaliações, coletadas após 12 horas, 18 horas e 24 horas da transição, respectivamente, terão peso de 6/24.

Os objetivos secundários são: estimar a associação entre a taxa de adesão ao VC baixo durante ventilação espontânea e a sobrevida; estimar a incidência de assincronia nas primeiras 24 horas após a transição para a ventilação espontânea e a associação com mortalidade; estimar a proporção de pacientes que retornaram à sedação e à VM controlada nas primeiras 24 horas ou em qualquer momento após a transição para a ventilação espontânea, avaliar a adesão à tabela de pressão positiva expiratória final (PEEP) por fração inspirada de oxigênio (FiO_2_) baixa nas primeiras 24 horas após a transição para a ventilação espontânea e identificar as barreiras associadas à não adesão à ventilação com VC baixo durante a ventilação espontânea.

### Critérios de inclusão

Faremos uma triagem de todos os pacientes admitidos nas UTIs participantes sob VM e incluiremos os pacientes que atenderem aos critérios de inclusão (Figura 1).

**Figura 1:** Diagrama de fluxo do estudo.

UTI - unidade de terapia intensiva; P/F - relação da pressão parcial de oxigênio pela fração inspirada de oxigênio; VM - ventilação mecânica; ECMO - oxigenação por membrana extracorpórea.

Os critérios de inclusão e exclusão estão descritos na tabela 1.

**Tabela 1:** Critérios de inclusão e exclusão de participantes

Critérios de inclusão
• Idade > 18 anos
• Pacientes com IRpA hipoxêmica, definida como PaO_2_/FiO_2_ ≤ 300mmHg, sob VM invasiva controlada, com duração prevista de VM de pelo menos 24 horas
• Pacientes em transição para ventilação espontânea (PSV, PAV Plus, NAVA, CPAP e APRV)
**Critérios de exclusão**
• Pacientes com traqueostomia
• Decisão de não manter ou adicionar medidas de suporte à vida no dia da avaliação (cuidados paliativos)
• Pacientes em ECMO
• Pacientes com fístula aérea ou barotrauma que impeça o monitoramento adequado do volume corrente
• Lesões graves no sistema nervoso central que resultem em um *drive* respiratório abolido ou muito elevado, para os quais não seja possível manter um volume corrente protetor

IRpA - insuficiência respiratória aguda; PaO_2_/FiO_2_ - relação entre pressão parcial de oxigênio e fração inspirada de oxigênio; VM - ventilação mecânica; PSV - ventilação com pressão de suporte; PAV - ventilação com assistência proporcional; NAVA - assistência ventilatória ajustada neuralmente; CPAP - pressão positiva contínua nas vias aéreas; APRV - ventilação com liberação de pressão nas vias aéreas; ECMO - oxigenação por membrana extracorpórea.

### Cálculo do tamanho da amostra

Não há estudos anteriores que tenham estimado a taxa de adesão ao VC baixo em pacientes com ventilação espontânea; portanto, usamos uma abordagem conservadora. Para estimar a prevalência com um limite de confiança de 5% de precisão, uma adesão antecipada de 50% e até 10% de perda de seguimento, estimamos que precisaríamos incluir 422 pacientes. Além disso, calculamos o poder de detectar uma diferença de 3 dias nos dias livres da VM, com desvio-padrão de 9 dias,^(22)^ e prevemos poder estatístico de, pelo menos, 80%, mesmo com taxa de perda de seguimento de 10%, desde que a prevalência esteja entre 25 e 75%.

### Medidas do estudo e coleta de dados

As seguintes características basais relacionadas à admissão na UTI e ao início da VM serão obtidas dos prontuários médicos do paciente: idade, sexo, altura (avaliada por meio de uma fita métrica com o paciente em posição supina), peso informado pela equipe da UTI, principal motivo de admissão na UTI, causa da IRpA, relação entre pressão arterial de oxigênio e fração inspirada de oxigênio (PaO_2_/FiO_2_) no dia do diagnóstico de IRpA, data da intubação, fatores de risco da SDRA, comorbidades (cardíacas, respiratórias, neurológicas, renais, hepáticas, imunossupressão, câncer, hipertensão arterial, diabetes e tabagismo) e *Simplified Acute Physiology Score 3* (SAPS 3). Calcularemos o PCP com as seguintes fórmulas:


$$\begin{array}{c} 50+0,91\times \left(\text{altura em centímetros}-\;152,4\right)\text{para homens}\\ 45,5+0,91\times \left(\text{altura em centímetros}-152,4\right)\text{para mulheres} \end{array}$$

Também coletaremos retrospectivamente os parâmetros ventilatórios do dia anterior à inclusão no estudo a partir dos prontuários médicos do paciente, além do uso de vasopressores, sedativos, bloqueadores neuromusculares e analgésicos, diagnóstico de SDRA, escore da Escala de Richmond de Agitação-Sedação (RASS - *Richmond Agitation-Sedation Scale*), presença de *delirium* avaliado pela escala *Confusion Assessment Method in an Intensive Care Unit* (CAM-ICU) ou avaliação clínica, escore *Sepsis-related Organ Failure* (SOFA), análise de gasometria e outros dados laboratoriais para diagnóstico de falência de órgãos. Também calcularemos a complacência e a resistência do sistema respiratório no dia anterior à inclusão no estudo (para pacientes sob sedação profunda e/ou bloqueio neuromuscular). A tabela 2 apresenta as principais medidas do estudo e os métodos de coleta de dados.

**Tabela 2:** Características da população do estudo

Característica		Valor
Idade (anos)		xx ± x
Masculino		n (xx)
IMC (kg/m^2^)		xx ± x
SAPS 3		xx ± x
SOFA		xx (xx - xx)
LIPS		xx (xx - xx)
Índice de comorbidades de Charlson		xx (xx - xx)
Causa da IRpA		
	Pneumonia (bacteriana, viral, oportunista)	n (xx)
	Sepse extrapulmonar	n (xx)
	Coma/rebaixamento do nível de consciência	n (xx)
	Edema pulmonar cardiogênico/insuficiência cardíaca	n (xx)
	Aspiração	n (xx)
	Choque hipovolêmico	n (xx)
	Outras	n (xx)
Dias de intubação antes da inclusão		xx (xx - xx)
Causa da hospitalização		
	Insuficiência respiratória aguda	n (xx)
	Sepse	n (xx)
	Doenças neurológicas	n (xx)
	Monitoramento pós-operatório	n (xx)
PaO_2_/FiO_2_ no diagnóstico de IRpA		xx ± x
Duração da VM invasiva (dias),		xx (xx - xx)
Dias livres da VM (dias)		xx (xx - xx)
Tempo de internação na UTI (dias)		xx (xx - xx)
Sobrevida na UTI		n (xx) (xx- xx)
Tempo de internação hospitalar (dias)		xx (xx - xx)
Sobrevida hospitalar		n (xx) (xx- xx)

IMC - índice de massa corporal; SAPS 3 - *Simplified Acute Physiology Score 3*; SOFA - *Sequential Organ Failure Assessment*; LIPS - *Lung Injury Preditivo Score*; IRpA - insuficiência respiratória aguda; PaO_2_/FiO_2_ – relação entre pressão parcial de oxigênio e fração inspirada de oxigênio; VM - ventilação mecânica; IIQ - intervalo interquartil; UTI - unidade de terapia intensiva. Os dados são expressos como média ± desvio-padrão, n (%), mediana (intervalo interquartil) ou n (%) (IC95%).

### Coleta de dados no dia da transição para a ventilação espontânea

No dia da inclusão no estudo, coletaremos parâmetros ventilatórios 1 hora antes da transição para a ventilação espontânea e periodicamente durante as primeiras 24 horas de ventilação espontânea. Incluiremos os pacientes que passaram pela transição e permaneceram por, pelo menos, 1 hora em um dos seguintes modos ventilatórios: ventilação com pressão de suporte (VPS), pressão positiva contínua nas vias aéreas (CPAP - *continuous positive airway pressure*), ventilação com liberação de pressão nas vias aéreas (APRV - *airway pressure release ventilation*), assistência ventilatória ajustada neuralmente (NAVA - *neurally adjusted ventilatory assist*), ventilação com assistência proporcional (PAV+) ou assistência ventilatória adaptativa (AVA).

### Uma hora antes da transição

Os seguintes dados sobre ventilação controlada serão registrados retrospectivamente: modo ventilatório; frequência respiratória programada; frequência respiratória total do paciente; volume minuto; pico de pressão nas vias aéreas; VC e PEEP. Também coletaremos sinais vitais (frequência cardíaca, pressão arterial média, saturação periférica de oxigênio), dados de gasometria arterial (obtidos o mais próximo possível da coleta de dados ventilatórios), presença de assincronias importantes,^(23)^ uso de vasopressores, uso de sedativos e analgésicos, pontuação da RASS, presença de *delirium* avaliada pela escala CAM-ICU ou a avaliação clínica.

### Uma hora e a cada 6 horas até 24 horas após a transição para a ventilação espontânea

Assim que o paciente for incluído no estudo, coletaremos prospectivamente os dados ao fim da primeira hora e a cada 6 horas (com tolerância de ± 1 hora) após a transição, incluindo o modo ventilatório, a frequência respiratória do paciente, o volume minuto, o pico de pressão nas vias aéreas, o VC, a PEEP, o ajuste de sensibilidade, a pressão de oclusão das vias aéreas em 100ms (P 0,1) e a presença de assincronias. Também coletaremos sinais vitais, análise de gasometria arterial (se houver), dados sobre o uso de vasopressores, dados do uso de sedativos e analgésicos e dados dos níveis de sedação medidos pela RASS e a presença de *delirium* (Tabela 3).

**Tabela 3:** Dados do dia da inclusão no estudo

Variável	1 hora antes	1 hora depois	6 horas depois	12 horas depois	18 horas depois	24 horas depois
Volume corrente (mL/kg de PCP)	xx ± xx	xx ± xx	xx ± xx	xx ± xx	xx ± xx	xx ± xx
PEEP (cmH_2_O)	xx ± xx	xx ± xx	xx ± xx	xx ± xx	xx ± xx	xx ± xx
Frequência respiratória (respirações/minuto)	xx ± xx	xx ± xx	xx ± xx	xx ± xx	xx ± xx	xx ± xx
Pico de pressão, (cmH_2_O)	xx ± xx	xx ± xx	xx ± xx	xx ± xx	xx ± xx	xx ± xx
Volume minuto (litros/minuto)	xx ± xx	xx ± xx	xx ± xx	xx ± xx	xx ± xx	xx ± xx
FiO_2_	xx ± xx	xx ± xx	xx ± xx	xx ± xx	xx ± xx	xx ± xx
pH	xx ± xx	xx ± xx	xx ± xx	xx ± xx	xx ± xx	xx ± xx
PaO_2_ (mmHg)	xx ± xx	xx ± xx	xx ± xx	xx ± xx	xx ± xx	xx ± xx
SaO_2_	xx ± xx	xx ± xx	xx ± xx	xx ± xx	xx ± xx	xx ± xx
Frequência cardíaca (bpm)	xx ± xx	xx ± xx	xx ± xx	xx ± xx	xx ± xx	xx ± xx
Pressão arterial média (mmHg)	xx ± xx	xx ± xx	xx ± xx	xx ± xx	xx ± xx	xx ± xx
RASS	x (x - x)	x (x - x)	x (x - x)	x (x - x)	x (x - x)	x (x - x)
*Delirium*	x (x)	x (x)	x (x)	x (x)	x (x)	x (x)
Assincronia	x (x)	x (x)	x (x)	x (x)	x (x)	x (x)
Vasopressores	x (x)	x (x)	x (x)	x (x)	x (x)	x (x)

PCP - peso corporal previsto; PEEP - pressão positiva expiratória final; FiO_2_ - fração inspirada de oxigênio; pH - potencial de hidrogênio; PaO_2_ - pressão parcial de oxigênio; SaO_2_ - saturação de oxigênio do sangue arterial; RASS Escala de Richmond de Agitação-Sedação. Os dados são expressos como média ± desvio-padrão, mediana (intervalo interquartil) ou n (%).

### Seguimento

Os pacientes serão seguidos até a alta hospitalar ou até o óbito dentro do hospital, e serão computados: a taxa de adesão ao VC baixo, ou seja, VC ≤ 8mL/kg de PCP nas primeiras 24 horas de ventilação em ventilação espontânea, a proporção de pacientes com assincronia significativa na transição para a VM espontânea, a proporção de pacientes que retornam à sedação e à VM controlada nas primeiras 24 horas, a proporção de pacientes que retornam à sedação e à VM controlada em qualquer momento durante a VM, a taxa de adesão a uma tabela baixa de PEEP/FiO_2_ nas primeiras 24 horas, a sobrevida hospitalar e os dias livres da VM 28 dias. Os dias livres da VM serão calculados como o número de dias vivo e fora da VM até o dia 28. Os pacientes que morrerem antes do dia 28 serão considerados como tendo zero dia livres da ventilação; o dia da inclusão será considerado o dia zero; nos pacientes que forem extubados e reintubados nos primeiros 28 dias, o número de dias livres da VM será calculado como o número de dias com vida e sem VM do dia da última extubação com sucesso (> 48 horas sem VM) até o dia 28; para pacientes com traqueostomia; o número de dias livres da VM será calculado como o número de dias vivo e sem VM a partir do dia em que o paciente permanecer desconectado do VM por mais de 6 horas consecutivas até o dia 28.

O cronograma de coleta de dados é mostrado na figura 2 e na tabela 1S (Material suplementar).

**Figura 2:** Cronograma da coleta de dados.

VM - ventilação mecânica; UTI - unidade de terapia intensiva.

### Análise do plano estatístico

Realizaremos estatísticas descritivas, usando a média e o desvio-padrão de variáveis contínuas que apresentem distribuição normal, a mediana e os intervalos interquartis de variáveis contínuas que não apresentem distribuição normal e proporções de variáveis categóricas.

As diferenças entre os pacientes que receberam VC menor ou igual a 8mL/kg de PCP e os pacientes que receberam mais de 8mL/kg de PCP nas primeiras 24 horas após a transição para a ventilação espontânea serão avaliadas por meio de testes *t*, testes de Mann-Whitney ou testes do qui-quadrado, dependendo das características do paciente e da distribuição dos dados.

Serão apresentadas: prevalência de adesão à ventilação com VC baixo na ventilação espontânea durante as 24 horas iniciais (VC menor que 8mL/kg de PCP), proporção de assincronia nas primeiras 24 horas após a transição para a ventilação espontânea, proporção de pacientes que retornam à sedação e à VM controlada nas primeiras 24 horas ou em qualquer momento após a transição para a ventilação espontânea e adesão a tabela de PEEP/FiO_2_ baixa nas primeiras 24 horas após a transição para a ventilação espontânea, com os respectivos intervalos de confiança de 95%.

As possíveis barreiras associadas à não adesão (VC durante as primeiras 24 horas de ventilação espontânea inferior a 8mL/kg de PCP) serão analisados por meio de um modelo de regressão logística.

O modelo de riscos proporcionais de Cox e as curvas de Kaplan-Meier serão usados para caracterizar a sobrevida hospitalar em 28 dias com base no VC durante as primeiras 24 horas de ventilação espontânea. Analisaremos a mortalidade em 28 dias usando o VC durante as 24 horas iniciais de ventilação espontânea e conforme a incidência de assincronia durante as primeiras 24 horas após a transição para a ventilação espontânea, usando regressões logísticas mistas com o centro tratado como um efeito aleatório.

A comparação do VC durante as primeiras 24 horas de ventilação espontânea (menor que 8mL/kg de PCP *versus* maior que 8mL/kg) em relação aos dias livres da VM em 28 dias será realizada por modelos mistos de regressão generalizada, considerando a distribuição que melhor se ajusta aos dados, incorporando o centro como efeito aleatório. Construímos um diagrama causal conceitual no formato de um gráfico acíclico dirigido,^(24)^ incluindo a associação entre o VC durante as primeiras 24 horas de ventilação espontânea e os dias livres da VM em 28 dias e as variáveis mais relevantes (Figura 1S - Material Suplementar): índice de massa corporal (IMC) e SAPS 3 basal, SOFA do dia anterior à transição, PaO_2_/FiO_2_ no momento do diagnóstico de IRpA e PEEP, RASS e pH 1 hora antes da transição.

Todas as análises serão realizadas usando o programa estatístico R (R Core Team; Viena, Áustria; https://www.R-project.org), e consideraremos p < 0,05 para indicar significância estatística.

### Coleta de dados e avaliação da qualidade

Todos os dados serão coletados por um pesquisador treinado de cada centro em um formulário eletrônico (eCRF - *electronic case report form*) desenvolvido no banco de dados eletrônico *Research Electronic Data Capture* (RedCap). O banco de dados eletrônico tem seus campos formatados para aceitar faixas específicas de valores relativos a cada variável, reduzindo as chances de inserção de valores incorretos. As variáveis mais importantes serão analisadas quanto a dados ausentes, discrepantes ou inconsistentes. Periodicamente, será salva uma cópia de segurança do banco de dados, que será arquivada ao fim do estudo.

### Aspectos éticos

O estudo foi aprovado pelo Comitê de Ética em Pesquisa do Hospital das Clínicas da Universidade de São Paulo (CAAE 28482720.0.1001.0068) e pelo Comitê de Ética de cada instituição participante, caso necessário. Dispensou-se o Termo de Consentimento Livre e Esclarecido devido à natureza observacional do estudo. O protocolo está registrado na plataforma internacional ClinicalTrials.gov (NCT06042036). Cada paciente será identificado por meio de um número de estudo, para proteger a confidencialidade.

Enviaremos os resultados do estudo para publicação seguindo as diretrizes recomendadas ao relatar estudos observacionais, *The Strengthening the Reporting of Observational Studies in Epidemiology* (Strobe).^(25)^ Os resultados do estudo primário serão publicados em um periódico revisado por pares.

### Situação atual

O recrutamento de participantes começou em junho de 2023, e esperamos concluir este estudo até junho de 2024.

## DISCUSSÃO

O estudo SPIRAL foi elaborado para descrever a ventilação de pacientes com IRpA em ventilação espontânea, principalmente com relação ao VC aplicado nessa fase. Ensaios clínicos randomizados demonstraram que VC baixo e pressão de platô limitada durante a ventilação controlada reduzem a mortalidade e a lesão pulmonar induzida pelo ventilador,^(3,7)^ e as diretrizes de prática clínica recomendam o uso de ventilação protetora, definida como VC de 4 - 8mL/kg e pressão de platô abaixo de 30cmH_2_O.^(2,26)^ No entanto, na ventilação espontânea, o VC varia conforme o nível ajustado de assistência inspiratória e o esforço inspiratório do paciente, o que dificulta o controle do VC. Estudos fisiológicos anteriores^(27,28)^ mostraram que é possível manter o VC abaixo de 8mL/kg de PCP na maioria dos pacientes diagnosticados com SDRA que estão fazendo a transição para a ventilação espontânea. O foco do estudo na aderência ao VC baixo durante a ventilação espontânea reflete os desafios enfrentados pelos médicos na prática diária. Optamos por usar um ponto de corte de 8mL/kg de PCP, em vez de 6mL/kg, porque, embora a maioria dos estudos clínicos randomizados tenham como alvo 6mL/kg de PCP, VC até 8mL/kg foram aceitas na maioria dos estudos.^(4,7)^ Além disso, as orientações clínicas definem a ventilação protetora como VC de 4 a 8mL/kg^(2,26)^ e estudos observacionais anteriores^(19)^ usaram essa definição.

Os objetivos primários e secundários do estudo abrangem um amplo espectro de resultados clínicos, incluindo dias livres da VM, sobrevida, incidência de assincronia e identificação de possíveis barreiras à adesão ao VC baixo, permitindo uma visão abrangente do efeito das estratégias de ventilação nos resultados centrados no paciente. Os dados serão coletados em vários momentos durante as primeiras 24 horas após a transição para a ventilação espontânea. Essa abordagem leva em conta possíveis variações ao longo do tempo e permitirá uma compreensão diferenciada da dinâmica do VC durante esse período crítico. Devido às possíveis variações no VC com a ventilação espontânea ao longo do tempo, optamos por usar a média ponderada do VC para obter uma avaliação mais representativa da dinâmica da ventilação durante as primeiras 24 horas de ventilação espontânea.

As características multicêntricas e multinacionais de nosso estudo, com a participação de dez países latino-americanos, acrescentam diversidade significativa à população de pacientes incluídos e permitem uma compreensão mais abrangente das variações regionais nas práticas clínicas. Este estudo observacional refletirá cenários clínicos da vida real, e os achados serão derivados de ambientes de assistência médica de rotina, aumentando a relevância prática dos resultados. Este estudo poderá influenciar a tomada de decisões clínicas, informar futuros ensaios clínicos e, por fim, melhorar os desfechos dos pacientes.

Nosso estudo tem algumas limitações. Coletaremos dados detalhados sobre os parâmetros ventilatórios somente nas primeiras 24 horas durante a transição para a ventilação espontânea e reconhecemos que essas primeiras 24 horas não são necessariamente representativas dos dias restantes na VM; além disso, os pacientes só podem ser incluídos uma vez no estudo, porque a sobrevida é um dos resultados secundários; portanto, coletaremos dados somente na primeira transição. O VC não será obtido continuamente, mas coletado em vários momentos durante as primeiras 24 horas de ventilação espontânea. Como forma de representar melhor essas variações, calcularemos a média ponderada do VC conforme a duração de cada período de avaliação. Esse método leva em conta as alterações no VC num período de 24 horas, atribuindo pesos diferentes a cada ponto de dados.

As UTIs participantes do estudo foram convidadas usando os bancos de dados de grandes redes de pesquisa, e, possivelmente, os pesquisadores das UTIs tinham mais conhecimento e experiência com VM do que a média dos profissionais das UTIs latino-americanas, o que poderia gerar viés de seleção e restringir a validade externa do estudo. Entretanto, os convites foram enviados a um grande número de UTIs, independentemente da experiência em pesquisa, e não restringimos a participação a membros ativos da rede. Embora a abordagem multinacional aumente a validade externa, as características regionais específicas das UTIs da América Latina podem limitar a generalização dos resultados para países de outras partes do mundo.

Em conclusão, este estudo pode fornecer contribuições valiosas para a compreensão das práticas de ventilação protetora em pacientes com IRpA durante a transição para a ventilação espontânea nas UTIs da América Latina, especialmente após a pandemia da COVID-19. Os resultados podem eventualmente informar a prática clínica e futuros ensaios clínicos e impactar o tratamento de pacientes com insuficiência respiratória aguda.
